# Illustrations of the Elementary Forms of Disease

**Published:** 1836-01

**Authors:** 


### Art. V.

*Illustrations of the Elementary Forms of Disease.* By Robert Carswell, m.d., Professor of Pathological Anatomy in the University of London.—*London.* Folio. 1833, 4, 5. Eighth Fasciculi. Longman.

We shall not apologize for devoting some pages to this work, notwithstanding it is already well known to the profession. It would be sufficient to remind our readers, were any excuse necessary, that the "Illustrations" are progressive, and that some parts of them are still very recent; but we have other and more important reasons for making them the subject of our consideration. Whilst it is our continual duty to observe and communicate every valuable novelty in medicine, it will occasionally be as much so to take a general survey of the science, and of its progress towards ideal perfection. In this employment it would be impossible to overlook Dr. Carswell's researches, the results of which may be reckoned among the most important achievements of British medicine in the present century. However agreeable it may be to watch the inpouring of new streams of wealth into the treasury of knowledge, it is to us no less pleasant, and certainly as useful an occupation, to count over the total amount of gains, to compare our present affluence with former indigence, and, above all, to consider in what manner our resources may be best disposed of, in order at once to ensure their multiplication, and to render them subservient to one great object of all science—the subjection of nature to art.

Though by no means desirous of setting out with mere verbal criticism, we cannot help expressing our doubts of the propriety of the title, "Elementary Forms of Disease." Could the author reveal even to our mental eye those mysterious shapes which, whether hidden by the general darkness of our ignorance, or too subtle in their essence to be apprehended by such instruments as we possess, or too changeable to be identified even when they have once been detected, have so often eluded the observation of philosophers, from the days of Hippocrates downwards, the whole medical world would stand immeasurably indebted to him. He would have gone far towards achieving for our science a discovery scarcely inferior to that which Bacon trusted, that in the fulness of time, his method of investigation would produce for all human knowledge,—the invention of "essential forms:"—" differentiæ veræ, sive naturæ naturantes, sive fontes emanationis!" But what expression could be found for our gratitude, were the author not only to pourtray these forms to our imaginations, but even to present them to the visual sense, by means of the familiar arts of drawing and engraving. The phrase "elementary form" can only be applied metaphorically, since disease, that is, disordered function, being a series of *events*, exists in time, not in space: it must therefore signify the initiatory event of the series. But is this likely to be discovered during our ignorance of the event which occupies a similar place in that series which constitutes the *un*disordered function. Few pathological conditions appear more susceptible of analysis than the hyperæmia of a mucous membrane; but till we can say how the blood, in permeating the tissue in its normal condition, observes a certain relation of quantity, and by what affinities it yields or assumes therein certain molecules and properties; till we can enumerate these events, and determine their order under circumstances of health, it will be presumptuous to profess an acquaintance with the primary change in a derangement of those actions. Perhaps it may seem that we are refining superfluously, and that the word disease was used to denote an alteration in the physical qualities of a structure, and that such and such kinds of disease, which make the subjects of our author's Illustrations, are denominated "elementary forms," only because they are less complicated than other forms, or because, though themselves composite, they do actually form the constituent elements of certain other diseases, standing indeed in the same relation to the latter as proximate principles to organic substances. In this sense tuberculous and scirrhous deposits, being essential components of a great variety of morbid growths, may be designated the elementary forms of the latter. But disease is a great abstraction, comprehending all diseases; and he who professes to treat of the elementary forms of them all, undertakes a task similar to that of discussing the proximate principles of all animal and vegetable substances.

We have seen no general plan of what Dr. Carswell proposes to do, but we judge that he will in due time favour us with illustrations of the essential characters of all kinds of morbid alterations of tissue to which the human frame is liable. It might therefore have been better to have entitled the work "Illustrations of the Elementary Forms of *Diseased Structure,*" which would comprehend not only what are commonly called organic, but also inflammatory diseases; and would exclude those in which the molecular changes are either unknown or incapable of pictorial representation.

Dr. Carswell's work belongs to one of the most interesting departments of pathological anatomy. This science may, we think, be said to include four different kinds of researches. The first to be mentioned, because of its priority in history, is that of which the object is the discovery of alterations in organs, *as organs,* and consequently with reference to the changes in their real or supposed functions during life; or, where the function is doubtful, with reference to the symptoms of the fatal malady. These objects of enquiry were the only ones that engaged the earlier morbid anatomists, and they have been diligently pursued in modern times, under the denomination of *special* pathology. Researches of this nature are obviously of the last importance in diagnosis and prognosis. The *second* species of morbid anatomy is that which examines the organs with reference to the tissues involved in diseases, and which traces in organs differing considerably from each other a similarity of lesion, determined by their possessing one or more tissues in common. This method of investigation, suggested by Bohn, practised in this country by Carmichael Smyth, and reduced by Bichat to a system which has immortalized his name, has been particularly useful in illustrating the affinities of diseases, —their local origin, their course, and their extension. The *third* species is that which looks for certain textural changes, common to a great variety of parts, and recognized by certain essential characters, which are only accidentally modified by the nature of the tissues themselves. Not to mention inflammation and its immediate products, the changes in question embrace deviations from the proper consistence of organs, excesses or defects of nutrition, perversions of secretion, and the deposition of peculiar substances. The last of these subjects has been particularly prosecuted by Abernethy, Bayle, Laennec, Andral, Cruveilhier, and Carswell. This division we consider the most important of all, because, as we shall see by and by, it conducts us nearer to the very beginnings of evil; or, in other words, assists us in ascending from local to constitutional causes, from diseases to diatheses,—an end of incalculable moment to prophylaxis. To the *fourth* species belong those investigations which trace original malformations, and subsequent transformations of tissues, and many other lesions, to the operation of laws which govern the processes of organization throughout the animal kingdom. Viewing it in this manner, we may recognize in a mal-formed organ the permanence of a condition which is usually confined to the embryonic state; or, in a diseased organ, the return to a lower type of development. Enquiries of this description teach us that a compound tissue, in its morbid transformations, can only assume the forms of which its elementary tissue is susceptible. Thus, fibrous tissue, cartilage, bone, skin, &c., can only be mutually convertible into one another, or *repeat* one another, or such structures as have cellular membrane for their fundamental tissue: they cannot be converted either into nervous or into muscular matter, nor these into those. This department of anatomy promises a harvest of most interesting facts, which, if not immediately applicable to practice, must throw great additional light upon the regularity of Nature's operations, and exhibit order and method in the midst of what might at first appear only chaos and confusion. But we hope for something beyond this. Should it be discovered that the anormal condition of an organ in the human subject resembles the normal condition in one of the lower animals, (as, for example, the lungs of aged persons resemble those of the chelonian tribe, more than those of young persons,) and that this condition is best maintained by certain modifications of the external stimuli of life, we may hence obtain suggestions respecting the circumstances that have favoured the degeneration in question. We need scarcely add, that this branch of anatomy, under the title of transcendental, has been zealously cultivated by Oken, Meckel, Geoffroy St. Hilaire, and Serres. The last-mentioned author has done more than any of his predecessors in connecting the anatomical discoveries with pathological doctrines.

The third division we have already said has nothing to do with particular organs or particular tissues; it is likewise unconcerned in the observation of analogies between anormal conditions of organs and their embryonic states in the same animal, or their normal conditions in other species. Its object is the investigation of particular pathological states, which may affect all or some only of the various organs and tissues. Thus, it searches for the essence of inflammation, and shews that it may be produced wherever there are capillaries and blood, and that it must be modified according to the composition of the latter and the arrangement of the former, &c. It likewise traces peculiar products to faults in the blood; which products may be discharged upon secreting surfaces, or may be deposited in place of the proper molecular structure of organs, and which will present certain forms, consistence, size, colour, &c., according to the nature of the tissue and other circumstances. Such pathological conditions correspond pretty closely to Dr. Carswell's Elementary Forms. Having said thus much by way of introduction, we shall proceed to consider the manner in which this able author has treated his subjects.

We may first mention that eight fasciculi have been published: the 1st, upon Tubercle; the 2d and 3d, upon Carcinoma; the 4th, upon Melanoma; the 5th, upon Softening; the 6th, upon Hæmorrhage; the 7th, upon Mortification; and the 8th, upon Pus. Each fasciculus contains four coloured plates, in which it is difficult to know whether to give more praise to the minuteness and fidelity of the representation, or to the beauty of the execution. The text is devoted partly to a general discussion of the subject, and partly to the explanation of the plates. In the latter so much pains have been taken, that we doubt whether, if the author himself were present at our inspection of the plates, he could indicate the several parts with more precision.

Before entering upon a critical analysis of the first fasciculus, we may perhaps be allowed to make one or two remarks upon the origin of morbid growths in general, without implicating the question, whether they depend upon disordered secretion, or upon disordered nutrition; nor shall we at present enquire whether, upon their first appearance, they assume the forms of cysts. A question of greater moment is, whether their cause is confined to the part in which they are found, or whether it must be sought in some fault pervading the whole economy. In favour of the former supposition, the following presumptions might be urged: 1st. The mere fact of the frequent circumscription of the diseased structure; since, if the cause were general, the effect might be expected to be so likewise.

2dly. The predilection which the morbid growths exhibit for particular organs; as tubercle for the lungs, scirrhus for the mamma and pylorus, encephaloid cancer for the testicle, &c. 3dly. The apparently close connexion between the anomalous formation and certain external agents, of which the operation is manifestly circumscribed: e.g. the production of scirrhus by a blow. 4thly. In certain cases, the apparently good health of the individual up to the commencement of the disease. 5thly. The direct ratio existing between the degree of the constitutional affection, and the activity of the local malady. Upon the first of these, it may be remarked, that we might admit the principle that it is superfluous to look for the cause of the alteration in a structure beyond its locality, were the materials of the structure derived exclusively from the place where the disease exists, or if all the structures in the body were so similar to each other as to forbid our supposing that the particular formation of some might occasion in them a more signal development of a fault more or less common to all. Assuming, for the sake of illustration, that the constitutional fault consisted in a defect of some proximate principle in the tissues generally, is it not all but certain that this defect would occasion more mischief in some organs than in others; those, namely, into which this principle most largely enters? or assuming that the evil was in the blood, we might rationally expect the composition of some structures or products to be more influenced than others, by an excess or deficiency of principles important to their very existence. There is consequently no more weight in the second argument than in the first of those above enumerated, since the greater frequency with which particular parts are affected would only indicate that the tissues of which they are composed, and the fluids which permeate them, are such as to be especially affected by a morbific cause, which prevails to a greater or less degree throughout the system; just as, in general hyperæmia, particular organs are apt to be more congested than others. In like manner we may dismiss the third argument, which proves nothing more than that a local agent may excite disease in an organ which was predisposed, but throws no light upon the mode in which the predisposition was brought about. With regard to the fourth, we may observe, that the cases in which there has been no manifest declension of health prior to the appearance of the morbid growth are very rare, and that, when they do occur, it will be found that some local cause has occasioned, in the part affected, a condition favourable both to the production and to the acceleration of the morbid process. Thus, we occasionally observe a very rapid tuberculization of the lungs supervening on an apparently good state of health; but in such cases the disease almost always begins with an attack of pneumonia or of bronchitis, which places the organ in circumstances highly favourable to the formation of tubercles. Or, to take the case of scirrhus of the mamma, occurring in a person apparently free from disorder; in the majority of instances, as every one knows, this affection is preceded by a change of complexion, by debility, and other signs of deteriorated health; but, in such as we have alluded to, it will generally be found to be the result of some accidental circumstance which occasioned an accumulation of blood in the organ, a blow, for instance, or exposure to a current of cold air. The fact adduced in the fifth presumption may be applied conversely: that is, the activity of the local disease may be considered only a sign of the intensity of that constitutional taint, the existence of which is attested in other parts of the body.

Very little doubt can rest upon our minds respecting the point at issue, if we now take into consideration the direct presumptions in favour of the supposition that morbid growths owe their origin to the state of the whole system. 1. The very frequent diffusion of the morbid matter in several organs at the same time; this diffusion not admitting of explanation upon any supposition of absorption. 2. The production of the substance in other parts of the body, after the removal of the organ in which it first appeared. 3. The well-known fact that a slight injury, which in most persons would produce nothing more than inflammation, and its usual consequences, will, in a particular subject, be followed by one of the diseased growths under discussion.

We have made these observations, because Dr. Carswell, in his Essay on Carcinoma, is so convinced of that for which we have been contending, that he contents himself with stating it as a law, "that the speciality of the morbid product is independent of any local agency whatever," without adducing any further proof of it than the fact which we have last adverted to. We could have wished, indeed, that he had discussed the question more fully; partly because we cannot doubt that he would have placed the arguments in a striking point of view, and partly because we are aware that there are some eminent pathologists not as yet prepared to acknowledge the law laid down. Dr. Hodgkin, for instance, expresses some scepticism as to the existence of the constitutional taint;* and Dr. Alison, while attributing tubercle, encephaloid and melanosis to a diffused constitutional peculiarity, arranges scirrhus and cancer among the formations which have a local origin.†

Assuming, then, that the heterologous formations are not of local origin, we have next to enquire whether the constitutional peculiarity belongs to the blood, or to the tissues, or to both. It needs no argument to prove that blood of a good quality may be sent to an organ, and yet that the nutrition and secretion in this part may prove very vitiated, in consequence of some failure of innervation, or of a derangement of those complicated affinities which preside over capillary changes. But, if the same kind of substance be produced in tissues of a very different organization, as, e. g., upon mucous and upon serous surfaces, in the coats of the stomach, and in

* Med.-Chirurgical Transactions, vol. xv. p. 321.
† Outlines of Physiology and Pathology, p. 551.

the substance of the brain, it is infinitely more probable that the deposit is derived from a common fluid which pervades them all, than that similar derangements should occur in tissues so dissimilar.

This *a priori* argument is strongly confirmed by the fact, that the substances in question are often formed in the blood itself, sometimes in the vessels, sometimes when effused. Dr. Carswell states, that carcinomatous matter is found not only in veins which ramify in the immediate vicinity of tumours, and which might be supposed to have derived it by absorption, but also in remote veins; and in one, in particular, which completely precludes the idea of absorption, viz. the vena portæ. Since there is so strong a tendency, then, in the particles of the blood to arrange themselves in the form of the substance alluded to, it is scarcely necessary to suppose any derangement in any of the other elements of nutritive and secretory processes, were it not that one part is often more affected than another, though of similar organization. Thus, a tuberculous or scirrhous deposit may be confined to one kidney, or to one hemisphere of the brain. In such cases we are disposed to think that very slight differences of mechanical condition, such as a temporary congestion, might be adequate to account for the fact, without supposing any special derangement of nutrition or of secretion.

How the blood becomes thus vitiated,—whether by defects in the assimilative, respiratory, and excretory functions,—defects produced by an originally faulty organization of the parts to which they belong,—or whether it only shares a fault inherent in the chemical composition of the whole system, are questions which we are not at present in a condition to solve.

In the author's description of the characters of Tubercles, the observation most worthy of attention is, that he reckons it among the inorganizable products of the system, and denies that it is in its first stage semi-transparent. In this respect his account agrees pretty closely with that of Andral, and differs from that of Laennec. The last-mentioned author evidently treats of tuberculous matter as if it were a *tissue*, or a part of the animal in which it is found. According to his views, it is an imperfect condition of the parenchyma, the result of an aberration of nutrition; it grows, as he expressly states, by intus-susception, and the changes which it suffers are the effects, not of any neighbouring agency, but of processes confined to itself. There was something in this view that at first appeared remarkably consonant with analogy. That in a cachectic state of the system, and consequently of the blood, the structure of a part should *degenerate*, (a term once so common, but now becoming obsolete in our schools of pathology;) that the vitiated structure should undergo spontaneous changes, such as becoming opaque and firm, and then soft and liquid; that it should be evacuated, and leave behind it a cavity; seemed to be not only highly probable *a priori*, but established by a careful observation of the actual facts of the case. Further researches, however, have thrown great doubt over Laennec's doctrines on this subject. Tuberculous matter is not a substitute for the textural particles of the organs in which it occurs, though it may ultimately interfere with their secretion, as we shall see presently. Its seat is not in the substance of the elementary tissues, and it presents no traces of areolæ, fibres, or canals, that would justify us in considering it an organized substance: nor was Laennec's description of the alterations in its qualities so correct as he supposed. Thus, the matter never itself appears first in a transparent form, though it is often accompanied by a transparent secretion; and Andral long ago perceived that, so far from the softening process beginning at the centre, it generally commences at the circumference, in consequence of the secretion of pus which it has provoked by its action as a foreign body.

Pathologists in the present day are agreed, then, that tubercle is an unorganized product; but concerning the mode of its deposition their opinions are more at variance. Cruveilhier holds that tubercle, like all other heterologous matters, is secreted in the areolæ of the cellular membrane, and at the expense of the nutrition of the organ; and that the proper tissue is atrophied. Andral considers it a perversion of that perspiratory secretion which, according to him, occurs wherever there is living matter. Dr. Carswell evidently regards it as an *excretion*, though he does not make use of that very term. We are not aware that any pathologist has made so many important observations upon tubercle as this author, and some of the most interesting of them have reference to its locality. He has satisfied himself that the seats are the blood, and mucous and serous surfaces, (the latter including the cavities of the cellular membrane,) but that mucous surfaces are the places where it most abounds. Upon this particular point his observations are entirely original, and are evidently based upon very careful anatomical investigations.

Tubercle, as every one at all acquainted with the subject is well aware, is very frequently found upon the surface of the pleura and of the peritoneum; but our author's researches have led him to the conclusion that, "in whatever organ the formation of tuberculous matter takes place, the mucous system, if constituting a part of that organ, is, in general, either the exclusive seat of this morbid product, or is far more extensively affected with it than any of the other systems or tissues of the same organ. Thus, the mucous system of the respiratory, digestive, biliary, urinary, and generative organs, is much more frequently the seat of tuberculous matter than any other system or tissue which enters into the composition of these organs." His discovery that the mucous membrane of the lungs is the seat of the deposit, affords direct and valuable assistance in explaining certain facts regarding the form and the changes of pulmonary tubercles. The definite rounded form of tuberculous deposit in certain organs, particularly the liver and the brain, has, we doubt not, contributed to the notion that it is an organized substance. But this circumstance is purely accidental, being determined altogether by the mechanical conditions of the part in which it occurs. Subjected to equable pressure, as in the organs which have been just mentioned, its form is globular: it affects a similar figure, though for different reasons, when secreted in a shut sac, or in a cell, whether an air-cell, or a cavity in the cellular tissue. In tubes, it takes different shapes, according to their distribution: in hollow viscera, as the uterus, and in the pelvis of the kidney, it moulds itself to their interior; and, "upon the surface of serous membranes, whether natural or accidental, it may have either a globular or lancinated form, as the secretion in which it originates may have taken place in distinct points, or from a continuous surface of greater or less extent."

It may not be amiss to remind our readers that a few years ago, when Cruveilhier's experiments on the production of tubercles, by the injection of mercury into the lungs, were repeated in this country by Kay and others, a good deal of stress was laid upon the globular form of the deposit found in the parts which the metal had reached, as indicating their identity with tubercles, and consequently supporting the doctrine of the inflammatory origin of these bodies. We repeated the experiments, and, though obtaining the same results, were unable to deduce any argument from the figure of the bodies in question, in favour of the supposition that they were tubercular; since the figure was precisely what might have been expected from the secretion of a common inflammatory product around the irritating bodies, namely, the *globules* of the metal.

Our author's mode of accounting for the transparent appearance which tuberculous matter so often puts on in the lungs and in the peritoneum, deserves particular notice. We have already mentioned that Laennec was of opinion that it *always* has this character when newly formed; and Louis entertains the same idea: and yet, as Dr. Carswell observes, with great force, this transparent substance is never seen elsewhere than in the lungs and on the free surface of serous membranes; and in these parts it certainly does sometimes precede the opaque matter. The cause of this appearance is assigned by Dr. C. to the simple fact, that the tuberculous secretion is mixed with the other secretions of the parts alluded to. The explanation cannot be better given than in his own words. Speaking of the air-cells, he says,

"The mucous secretion of their lining membrane accumulates where it is formed; but it is not pure mucus; it contains a quantity of tuberculous matter mixed up with it; which after a certain time is separated, and generally appears in the form of a dull, yellow, opaque point, occupying the centre of the grey, semitransparent, and sometimes inspissated mucus. This process of separation of tuberculous matter from secreted fluids is strikingly exemplified in tubercular peritonitis. When we examine the peritoneum thus affected, the three following stages of the process are frequently extremely well marked: first, on one portion of this membrane there is seen a quantity of recently secreted coagulable lymph; secondly, on another we find the same plastic semitransparent substance, partly organized, and including within it, or surrounding a globular mass of tuberculous matter; and, lastly, on another part the coagulable lymph is found converted into a vascular or pale cellular tissue, covered by an accidental serous membrane, beneath which, and external to the peritoneal or original secreting surface, the tuberculous matter is seated, having the form of a round granular eminence, resembling in colour and consistence pale firm cheese." (Fasc. 1.)

The *consistence* of tuberculous matter may become firmer by absorption of the watery secretion which accompanied its deposit, as in the lymphatic glands; or may be softened by infiltration with the fluids which its presence has caused to be secreted. In the latter case, which is the most common, it may be converted into a fluid, of a colour corresponding to the matter with which it is mixed, whether blood, serum, or pus. The softening, then, if this be true, must begin at the *circumference.* But how does this agree with the observation made by Laennec, and confirmed by every pathologist of experience, that the centre of a tubercle is often found soft, though the other part has considerable firmness, or, to use another phrase, is in "a state of crudity." It must be remembered that this observation applies only to tubercles in the lungs; and, if our author's account of the manner in which the deposit is formed in these organs be correct, (and we see no reason to dispute it,) the fact admits of an easy explanation, without supposing that the softened spot indicates the commencement of a change peculiar to the substance in question. Thus, if the morbid product be secreted upon the mucous membrane of the air-cells and bronchial tubes, and not at all in the adjoining tissues, it may be so accumulated as to occupy the whole space of the cavity, *excepting a central portion, containing mucus or some other secreted fluid,* which consequently will give to the cell or the tube, when divided transversely, the appearance of a central depression, mistaken by Laennec for the beginning of the change from crudity to softness. This view reflects credit upon Dr. C.'s ingenuity, and appears to us to be subject to no other doubt than as to the probability of mucus remaining upon the surface of that other secretion which covers the secreting membrane. This difficulty, however, is easily got over, if we take into consideration that it is not necessary that the fluid should be the immediate product of the part in which it is found, and which it may have reached after escaping from other parts of the bronchial surface.

The encysted form which tubercles are said to assume in some cases, Dr. Carswell pronounces to be a deception, produced by the dilatation and distention of a cell, or a tube, in which the matter is deposited. We do not ourselves see why a cyst is not as likely to be formed around tuberculous as around any other foreign body. Our author, of course, admits that an accidental tissue is formed around the matter in the process of cure. His remarks upon the transformation of tubercle into a putty-like substance, by the absorption of the albuminous constituents, and by the condensation of the earthy particles, as well as those upon the changes in the surrounding tissue, are very interesting; but we must content ourselves with referring the reader to the work itself, and to the plates, in which these changes are illustrated with singular felicity.

In examining the plates, we particularly recommend the inspector not to content himself with merely looking, but to look under the direction of the remarks which explain the representation. Without the latter, even the best-instructed eye may overlook some very important particulars. Where all represent their originals so faithfully, it is difficult to single out the most excellent; but we have been particularly struck with the delineations of tuberculous matter deposited in the bronchi, and upon the peritoneal covering of a portion of intestine.

Under the head of *Carcinoma*, the author treats, with the hand of a master, of some of the most malignant diseases to which the human frame is liable. He offers at the outset a remark, to the truth of which we cordially subscribe; namely, that great obscurity has been thrown over heterologous diseases in general, by the circumstance of their having been in former times studied almost exclusively upon the surface of the body. Differences of form, of consistence, and of structure, depending in reality upon differences in the degree of development of the same substance, or in the nature of the tissue in which the substance is formed, have been mistaken for differences in the essential nature of the morbid products. More extended and rigorous examinations, however, have led to the discovery of primary forms common to several varieties; one of these is carcinomatous matter. That the diseases comprehended under Carcinoma have something in common, we cannot for a moment doubt, because, although differing greatly in physical characters, and even in their anatomy and physiology, they are all found to prevail in the same habit of body, to coexist in the same individual, and to pass into each other. In addition to these signs of affinity, Dr. C. observes that "they all terminate in the gradual destruction or transformation of the tissues which they affect;" and "all possess, although in various degrees, the same reproductive character."

The genus Carcinoma he divides into two species, which he designates Scirrhoma and Cephaloma. These names would imply only a difference of consistence; but he wishes it to be understood that the species are separated upon grounds of difference of a more constant character; viz. that the former have little or no tendency to organization, while the latter are very readily organized. He admits, however, that though, for the most part, the matter of scirrhoma bears no trace of organization, yet it sometimes is changed into matter which becomes organized; an admission which, we think, ought to have prevented him from making any distinction on the above grounds. We confess not to understand, at first sight, how a secreted substance is first inorganizable, and then becomes a substance which is capable of being organized. Dr. C., however, appears to use the phrase "capable of being organized," in the sense of vascularity; and, with this understanding, we see no objection to the classification, though we should certainly dissent from a view that implied that scirrhous matter was not an organized product; since there can be no doubt that it is often a substitute for the proper tissue of an organ; a fact which the author himself takes great pains to illustrate. Thus, in the stomach, it takes the arrangement of the muscular fibres, and in the liver the form of the acini; under which circumstances it must surely be considered an organized substance, though degenerate, and though not readily convertible into a substance of which the nutrition is independent of the organ in which it is formed. Were the latter circumstance to be considered the characteristic of organization, it might be denied even to the molecules, for which the carcinomatous matter is substituted. We are therefore disposed to view Andral's arrangement of Scirrhus among the organized products, as more correct than that of our author; though we do not the less incline to the belief that the substances ranked under the head of Carcinoma may be correctly distributed into two groups, characterized by their having a tendency or not to become *vascular*.

The Scirrhoma of Dr. Carswell includes scirrhus, the pancreatic sarcoma of Abernethy, the Tissue lardacé of many French authors, the Matière colloïde of Laennec, and the Cancer gélatiniforme, or aréolaire, of Cruveilhier. To Cephaloma belong the common vascular or organized sarcoma of Abernethy, and the medullary sarcoma of the same author, called by Laennec Matière cérébriforme, or encéphaloide; by Mr. Burns, Spongoid inflammation; by Dr. Monro, Milt-like tumour; and by others, Soft cancer. The Fungus hæmatodes of Hey and Wardrop, and the Fungoid Disease of Sir A. Cooper, are peculiar states of the last-mentioned formation. The author's definition of Carcinoma is as follows:

"From the general considerations into which I have entered on the species and varieties of carcinoma, it must be obvious that no precise definition can be given of this disease. It may, however, be said to consist in the formation or deposition of a peculiar substance, which presents great variety of consistence, form, and colour; frequently assumes a definite arrangement, and possesses a vascular organization of its own; gives rise to the gradual destruction or transformation of the tissues in which it is situated; affects successively or simultaneously a greater or less number of organs, and has a remarkable reproductive tendency." (Fasc. 2.)

Although there is some vagueness in this definition, as the proposer of it himself allows, it contains, if we mistake not, sufficient to enable us to diagnosticate it from the only other heterologous deposit with which it is likely to be confounded, viz. tubercle. Melanosis is, of course, out of the question; but from the former it may be distinguished by its occupying the molecular structure of organs, and by its tendency to become vascular.

The remarks upon the "seat and origin of Carcinoma," by which the author means the source of its formation, and the place of its deposition, are deserving of very attentive perusal; particularly the account of the manner in which it enters into the structure of organs: how, for instance, its presence in the liver is betokened by a change in the colour and in the consistence of the acini, while their form and bulk remain for a time unaltered; how, by the grouping of a number of acini, and by their subsequent increase of bulk, they lose their original form, and are transmuted "into a lardaceous mass, or into some one or other of the tissues or substances which belong to either of the two species of carcinoma;" how (to take another example,) the deposit in the stomach often begins to show itself by a paleness and unnatural firmness of the muscular fibres, in which it is the more easily recognized that the colour of the tunic differs from that of cellular tissue; and how, in process of time, they "both acquire a greater or less increase of bulk, become remarkably distinct, and present that fibriform arrangement, hardness, and transparency, which are considered characteristic of scirrhus."

These changes are admirably depicted in one of the plates belonging to this fasciculus, if we make the exception that the colouring appears somewhat too vivid. The description of the formation of the deposit upon serous surfaces, and the application of the facts under this head to the probable origination of the matter in the blood, are likewise highly valuable. The remarks upon the appearance of carcinoma in the blood-vessels are so interesting, that we shall present them entire.

"The presence of the heterologous substance which constitutes the several varieties of both species of carcinoma in the blood is a circumstance of great importance, and unless it be clearly demonstrated to arise in consequence of a modification of the blood itself,—in whatever manner produced,—we should find it impossible to explain many of the phenomena which it presents, more especially those which accompany its formation in the molecular structure of organs, and on the surface of membranes.

"The following facts may be adduced as furnishing strong evidence that the formation of this substance takes place in the blood, whether it be found in this fluid alone, or in other parts of the body at the same time; 1st, the presence of this substance in the vessels which ramify in carcinomatous tumours, or in their immediate vicinity; 2d, in the vessels of a portion or of the whole of an organ, to the former of which this substance is exclusively confined, and can be traced from the trunks into the branches and capillaries; 3d, in vessels having no direct communication with an organ affected with the same disease, as, for example, when it is confined to a small extent of the vena portæ; and lastly in blood which has been effused into the cellular tissue, and on the surface of organs. The divisions of the vascular system in which the carcinomatous substance has been observed are the venous and capillary,—a circumstance which may be ascribed to the contractile power of the arteries preventing, under ordinary circumstances, the blood from accumulating, and, consequently, this substance from forming within them, and not to any peculiarity of function exercised by the former. The appearances which the carcinomatous formation presents in the blood are very various, and sometimes perfectly similar to those which mark its presence in the substance or on the surface of organs. In large veins, such as the vena portæ and its branches, the emulgent vein, &c. it may present the lardaceous, mammary, medullary, or hæmatoid characters, all in the same venous trunk. These varieties of the carcinomatous formation may be found mixed together in minute quantities, or isolated into masses so conspicuous that we can readily distinguish them from one another; sometimes they lie merely in contact with the internal parietes of the vein; at other times they are united to the latter by means of a thin layer of colourless fibrine; or minute blood-vessels pass from the one into the other, and are often very numerous and remarkably conspicuous in the cerebriform matter. The *formation* of these varieties of carcinoma in the blood cannot remain a matter of doubt to those who have had occasion to examine the appearances I have described. That the presence of an organized product in the blood can have no other origin than the blood itself, and that such a product cannot be introduced into this fluid by absorption or otherwise, are facts so obvious that I shall not attempt any further illustration of them." (Fasc. 2.)

With the above views, the supposition of a peculiar modification of growth in the part where the morbid product is found would of course be incompatible. The matter is separated from the blood, and assumes a certain form, bulk, colour, and consistence, not by virtue of any inherent peculiarities of organization, but of the circumstances of its locality.* It is not a growth, germinating as it

* Since writing the above, we have examined a very striking case of carcinoma of the uterus, confined to the body and fundus. In the latter part, and in the posterior parietes, the morbid deposit resembled in colour and consistence a section of the white part of *brawn*. Where it projected into the cavity, and consequently suffered less pressure, it was softer, and had somewhat of a cauliflower appearance: in one part it was vascular and fungoid. The anterior wall of the organ was thinned, and partially ulcerated, by the pressure of the carcinomatous matter, which must have grown from behind forwards. But the most interesting appearance was a black discoloration of the serous surface, chiefly between the uterus and the rectum. We found it to be occasioned by a thin layer of extravasated blood under the peritoneum, which had most probably been blackened by the chemical agency of a very ill-conditioned fluid, that was contained in considerable quantity in the peritoneal sac. In the effused blood there were three patches of a dirty yellow substance, each rather larger than a sixpence, were from the coagulable part of the blood, as was taught by Abernethy, nor is it (in the language of Laennec) an adventitious tissue. Neither can it be said (with Dr. Hodgkin) to be always a cystiform structure. In commenting upon the views of the last-mentioned pathologist, as developed in his admirable essay* on Adventitious Structures, our author admits that, if cysts exist in any of the organs of a person who is the subject of the cancerous diathesis, they may become the seats of the peculiar deposit, equally with any other serous membranes. He observes, however, that not only is the carcinomatous secretion often produced where there are no cysts at all, but that, where they do exist, the matter is often secreted in the cellular tissue external to them, in such a manner that the serous membrane is projected before them; a fact which is actually represented in Dr. Hodgkin's paper. This view would reduce the relation between cysts and carcinomatous deposits to one of a very accidental character; much more so than we are disposed to admit, partly because we think it unlikely that, with such powers and opportunities of observation as are possessed by Dr. Hodgkin, he should have attributed to scirrhous formations in general an arrangement which, according to Dr. Carswell, occurs only when there chance to be cysts in the vicinity of the carcinomatous secretion. Though by no means believing that cysts are at all essential to the formations under discussion, we certainly think that they do take a part, though a subordinate one, in the production of the peculiar substance. Is it not probable that they are sources of irritation in their locality, and that the consequent afflux and congestion of blood in the surrounding tissues occasion the morbid deposit?—that, in fact, they operate in a manner analogous to that of a local injury? The non-occurrence of cysts in the liver, walls of the stomach, the lungs, kidneys, lymphatic glands, brain, spleen, and blood, during the development of carcinoma, seems a rational objection to the general application of the doctrine of the cystic origin of tumours; but we are not sure that Dr. Hodgkin would not consider this statement a *petitio principii*, and allege that, although simple cysts containing fluid may not be present, yet the very carcinoma itself, when properly dissected, would prove the existence of the structure which is denied; viz. that of a cyst, the cavity of which is filled up by a number of smaller cysts, having the form of pedunculated tumours, upon which the larger cyst is reflected, in the manner of a serous membrane. With cysts of this description there may not perhaps be coincident cysts of the more ordinary kind, and yet the former may not the less exist. Whether carcinoma can in all cases be unravelled into such a growth as that which Dr. H. has described, is another question, which can only be which we scarcely doubt was carcinomatous matter, separated from the blood. The extravasation we attributed to the congestion occasioned by the presence of the enlarged uterus upon the veins.

* Medico-Chirurgical Transactions, vol. xv.

determined by dissection, not by the presence or absence of cysts, such as we usually understand them.

We cannot leave this part of our subject without saying that, whether this theory be true or not, Dr. H.'s essay is extremely valuable in other respects; and particularly for a summary which it contains of the distinguishing characters of malignant diseases.

We can only present a very hasty abstract of Dr. Carswell's able description of the physical characters of Carcinoma. The three principal forms are, the Tuberiform, the Stratiform, and the Ramiform. The first of these is the most frequent and various, being in organs of equal density, globular; upon serous surfaces, passing from a globular to a pyriform shape, either on account of the mode of its attachment, or of less resistance being opposed to its growth in one direction than in another; fungiform, when resisted anteriorly, but free laterally; lobulated, when accumulated in separate portions of the cellular tissue; of a cauliflower or mulberry form in the sub-mucous cellular membrane, being in that part collected in separate cells, and but little constrained in its increase by the mucous tissue.

"That appearance of carcinoma which resembles the structure of the pancreas depends generally on the agglomeration of very small globular or pyriform tumours, separated from one another by cellular or cellulo-fibrous tissue, but inclosed in a common capsule." (Fasc. 3.)

The stratiform arrangement is met with chiefly in the subserous cellular tissue, the physical qualities of which sufficiently explain this distribution. We have traced it in the peritoneum, throughout the intestinal tube, as well as upon the abdominal parietes. In one case of this description which fell under our observation, the omentum was converted into a solid prism-shaped body, lying between the stomach and colon, flattened at one extremity near the spleen, and somewhat lobulated, so as to resemble the pancreas, for a disease and displacement of which it was at first mistaken, till that viscus was discovered in a tolerably healthy state. The ramiform arrangement takes place when the morbid matter is collected in the veins: this appearance is most frequently found in the kidney.

The *bulk* of carcinomatous formations is closely connected with the degree of pressure to which they are subjected. Their slow progress, when restrained by a fibrous membrane, and the frightful rapidity with which they increase where that obstacle is removed, are both exemplified in carcinoma of the eye, before and subsequently to the operation of extirpation. The bulk of these formations is not determined by physical causes only: in the cephalomatous species, the size and growth are mainly dependent on the vascular organization.

The *consistence* depends, 1st, upon the structure of the organ affected: thus, it is denser in the liver than in the brain, and in the skin than in the submucous cellular tissue, being subjected to greater pressure in the one situation than in the other: 2dly, upon the elementary composition of the deposit: thus, in the same stage of development, in the same organ, and with the same degree of pressure, as upon the surface of a sore formed by the destruction of the protruded portion of a tumour, (e. g. the bottom of the orbit after removal of the eye,) it will be found of very various density. 3dly. Upon certain changes in the matter itself and in the surrounding tissue, which we shall allude to directly.

The sections upon the chemical composition of Carcinoma, and upon its anatomical characters, will not admit of condensation. With regard to the latter, however, we must not omit to remark, that the accurate description of the structure of scirrhoma confirms the objections which we offered just now to the grounds upon which the author distinguishes this species of carcinoma. When he speaks of its containing cellular, fibrous, and serous tissues, and moreover takes some pains to prove that what Andral calls mere hypertrophy of the cellular membrane is in reality "a tissue *sui generis,* produced by the uniform distribution and molecular deposition of the carcinomatous matter, either in the cellular tissue of an organ, or in an accidental tissue of a similar kind, formed during the deposition of the carcinomatous matter," it seems surprising that he should have characterized it as indisposed to organization. That it does not become *vascular,* appears pretty certain; for, notwithstanding that vessels are occasionally perceptible, they will, upon careful examination, be found to belong to the tissues in which the deposit is formed. The description of the proper circulation of cephalomatous tumours, as distinguished from their collateral circulation, (Fasc. 3), is extremely graphic. In the course of some observations upon the physiological characters of carcinoma, the author shews satisfactorily enough how some of the most striking phenomena that occur during its progress are produced by alterations either in the proper or in the collateral circulation; a fact of considerable importance, inasmuch as it forbids our attributing, as we are but too apt to do, the changes in question to some specific processes peculiar to the morbid product. To take an instance: a mechanical congestion in the collateral veins is frequently the cause of such congestion in the whole or in a part of the tumour, as not only to alter its colour, but to produce hemorrhage, either internal or external. This occurrence may take place very early in the disease. We remember the case of a countryman who had a considerable tumour in the vicinity of the deltoid muscle, the origin of which he stated to have been almost sudden, after violent exertion with a flail. The abruptness of its appearance led many to think that some blood-vessel had accidentally given way during the muscular efforts; but the tumour proved ultimately to be genuine encephaloid, which, in all probability, had existed for some time prior to the violence, but had received a sudden accession of bulk by the occurrence of hemorrhage. Not only hemorrhage, but mortification, may result from the compression of veins: this compression being sometimes caused by smaller tumours in the neighbourhood of the formation, and sometimes by the unyielding nature of the tissue.

We extract the following account of the ulceration of carcinoma.

"It is well known that it is the most projecting part of a tumour situated beneath the skin in which a solution of continuity commences; and the reason of this is, that it is here the circulation is first arrested from the greater degree of compression to which the blood-vessels are subjected, together with the increased influx of blood caused by a greater degree of irritation. The most elevated portion of the skin becomes atrophiated during the first stage of compression and irritation, that is, when the circulation of the blood through it is only impeded; but so soon as this all-important function has ceased, which is announced by a change of colour from bright to dark red, purple, or black, a diminution of sensibility and temperature, it begins to soften, soon sloughs and exposes the subjacent portion of the tumour whose circulation had been similarly modified, softened, and deprived of vitality to a greater or less depth. The edges of the solution of continuity of the skin when first formed are sharp and irregular, they are not *everted*; they are, on the contrary, sometimes *inverted*; and their thickness is in proportion to the depth of the slough. The peculiarity of form assigned to the edges is produced by the subsequent development of the carcinomatous substance situated beneath them, which, being entirely freed from pressure all round their internal margin, necessarily projects forwards, as it grows, towards the centre of the tumour hollowed out by the softening and sloughing process, and, consequently, carries them gradually upwards and backwards. They acquire at the same time, a great accession of bulk, and form a rounded undulating border, beneath which the skin is found doubled upon itself, encircling the carcinomatous excavation." (Fasc. 3.)

We may remark, that Dr. Hodgkin's mode of accounting for the peculiar ulcer is in some respects similar to the above. Believing the whole formation to consist of several cystiform tumours growing from a common cyst, he states that, in the progress of growth, the central tumours are so compressed and strangulated, by the increasing bulk of those around them, as to lose their vitality; and thus to become the subjects of a softening and sloughing process. For the production of this effect, however, he thinks that the strangulation must be somewhat sudden: otherwise, the central bodies may be converted into a dense structure, and ultimately become penetrated by earthy particles. Such changes occur in the scirrhous tubercles of the liver and of the uterus.

All the changes incident to Cephaloma, viz. congestion, hemorrhage, softening, and sloughing, may occur also in Scirrhoma; but, in the latter, they depend on the vascular system of the tissues contained in the carcinomatous matter; in the former, upon the proper and collateral circulation.

Nerves have never been reproduced in Carcinoma. This we might, *à priori*, infer from our knowledge of the laws of organic development. Carcinoma, a deteriorated organization, could never present that which all analogies lead us to believe to be the *summum opus* of the *nisus formativus.*

*Melanoma* is not, as it might at first-sight appear, an accommodation of the terminal syllable of the more familiar designation to that of Carcinoma. It implies more than the melanosis of Laennec; for, whereas the latter was a name given to a morbid product, *sui generis,* the former is employed by Dr. Carswell to signify all "black discolorations or products," which he separates into two groups, the True and the Spurious. Upon the description of the former of these we do not think it requisite to enter, though it possesses a particular interest, by reminding us of some of the author's earlier researches, conducted in concert with one whose bright though brief career promised much to the pathology of his country. We allude to the late Dr. W. Cullen, who published a joint memoir with our author upon Melanosis, in the second volume of the Edinburgh Medico-Chirurgical Transactions. The source of this, as of the other morbid formations which have engaged our attention, Dr. Carswell traces to the blood, in which he is of opinion that there is an accumulation of the carbon usually employed in colouring the hair, the rete mucosum, the choroid, and other parts. It is a curious fact, and certainly a presumption in favour of this notion, that grey and white horses are much more subject to melanotic formations than the bay, black, or brown. Chemical analysis, and the anatomical distribution of the substance, certainly lead us to believe that it is derived from the blood: whether it be really identical with the matter usually appropriated to natural colours and pigments, we do not pretend to determine: for Dr. C.'s arguments we must refer the reader to the work itself.

Of the pseudo-melanotic formations, the first described by the author is that which occurs in the lungs, and which is produced by the introduction of carbonaceous matter. Mr. Pearson, and subsequently Laennec, had intimated that some kinds of pulmonary discoloration were owing to carbonaceous impregnation of the tissue; but the opinion was first put to the test of experiment, in a case published by Dr. J. C. Gregory, in the Edinburgh Medical and Surgical Journal, No. cix. Dr. Christison's analysis of the black matter contained in the lungs identified it with "the ordinary products of the distillation of coal."

"The physical characters of this form of spurious melanosis, viz. the uniform black colour of both lungs; the absence of any similar discoloration of any other organ; the occurrence of the disease in those habitually exposed to the inhalation of the coal-dust always contained in the atmosphere of a mine; and the black matter found in the lungs consisting essentially of this substance; are circumstances which demonstrate clearly the origin of the black matter, and its identity with the carbonaceous powder inhaled with the air in breathing." (Fasc. 4.)

Another species results from the operation of chemical agents on the blood. Of these, the most common are the acid contents, whether natural or morbid, of the alimentary canal. The discolorations vary "from a dull yellow orange tint to the colour of chocolate, bistre, or soot." The distribution is punctiform and ramiform when the blood acted upon is contained in the capillaries and veins of the stomach; and its extent is proportionate to the quantity and to the diffusion of the gastric acid, and its intensity to the quantity of blood contained in the vessels of that organ. The following observations are very interesting:

"The ramiform black discoloration of the blood from the pressure of an acid is seldom met with in the intestines, but the punctiform is not uncommon. The latter takes place in the capillaries of the villosities, and in those around the orifices and bases of the follicles. When the villosities are the seat of the discoloration, the surface of the mucous membrane appears as if it had been dusted all over with fine powdered charcoal, which gives to it a deep grey or slate colour. The discoloration may occupy the orifice of a number of contiguous isolated follicles, or of the aggregated follicles, and give rise to an appearance resembling acne punctata; or it may surround the orifice or basis of the isolated follicles, when it has an annular form.

"These appearances observed in the villous and follicular structures of the intestine have always been confounded with those produced by chronic inflammation. Both lesions are so similar in their physical characters, that it is only from the absence or presence of an acid in the affected portions of the intestine that we can form a decided opinion on the nature of either.

"The ramiform black discoloration of the blood is met with occasionally on the peritoneum, in cases of chronic tubercular peritonitis. The tubercles scattered over the surface of the peritoneum are surrounded by a dark ring, or a multitude of minute vessels, filled with black blood, either grouped close together, or having a stellated arrangement. The tubercles, if small, are thereby greatly obscured, and the peritoneum appears as if spotted with a deep brown, or black pigment." (Fasc. 4.)

The *stratiform* discoloration is most frequently seen either in the subserous tissue of the peritoneum, or in its accidental membranes when blood has been effused. The *liquiform* occurs in the stomach, when hemorrhage from any cause has taken place, and constitutes the black vomit of cancer. Perhaps the "coffee-grounds" vomit owns a similar cause. Melæna, and the black pitchy matter found upon ulcers of the large intestines, are, as every one knows, referrible to the action of sulphuretted hydrogen upon blood. Blood in the peritoneal cavity is sometimes blackened; in which case it is probable that the gaseous acid just mentioned is the agent, after penetrating the coats of the intestine.

Stagnation of the blood in the capillaries, or venous extremities, is sufficient to produce a spurious melanosis, without the co-operation of any chemical agent. The blackness is attributed by Dr. Carswell to coagulation, and to the escape of the serum, which carries away with it the saline particles, which there is good reason to believe have much to do with the red colour of the blood. In the lungs, this kind of discoloration constitutes the "matière noire pulmonaire," and may occur in any disease which is attended with much obstruction to the pulmonary circulation.

"The grey or bluish grey colour of the tuberculous matter so often met with, is occasioned by the presence of these black points, which appear to have their seats in the air-cells, the circulation in which is early impeded by the accumulation of the tuberculous matter within them." (Fasc. 4.)

The blackening of the bronchial glands is due to the same causes. It is most frequent in aged persons, but occasionally is found in young subjects affected with tubercular phthisis. We have found amid a cluster of these glands, enormously enlarged by tuberculous deposit, two or three smaller ones of a bluish black; in which it is possible that sanguineous obstruction had been occasioned by the morbid enlargement of the others, while the obstruction was perhaps a preventive of the secretion of the tuberculous matter to the same extent.

The dark discoloration which is found in the mucous membrane of the intestines, and particularly of the cæcum and colon, (designated by French writers "*coloration ardoisée*," is frequently the result of chronic inflammation; but it may be imitated by the action of acid matter upon the blood contained in the capillaries of the membrane. Dr. Carswell thinks that there is no other method of distinguishing the pseudo-morbid appearance than that of testing the presence of an acid. We conceive, however, that there would be but little use in this procedure when the agent has been sulphuretted hydrogen, notwithstanding it is a gaseous acid. The fallacies to which this coloration is likely to give rise were first pointed out, we believe, by MM. Kigot and Trousseau. In the present state of our knowledge, we must be content to decide upon the value of this sign, in much the same manner as upon that of *redness*; namely, by ascertaining whether it is accompanied or not by changes in the consistence or thickness of the tissue, or in the secretions of the part discoloured.

The subject of the fifth fasciculus is *Softening*. Were the term confined to that species of softening which is a perversion of nutrition, *sui generis*, there would be no objection to its arrangement among the "elementary forms;" but when we find ramollissement from inflammation, and from obliteration of arteries, placed under this head, it is impossible to help being struck with the inconsistency; more particularly as the author does not think it requisite to devote a special discussion to the idiopathic species. He remarks, however, in general terms, that softening may be the effect of a lesion of the blood, and may extend to the tissues generally.

"But there is another kind of this lesion, of a much more general character, and which is also very different in its nature from the two former. It occurs in almost all the textures of the body at the same time, although it may be so slight in some as hardly to be observable; whilst in others, even the hardiest, it may be strongly marked. It is never observed unless in individuals in whom nutrition in general is greatly modified. The modification of nutrition which precedes the softening process is, however, very different in kind in different individuals, a difference which obviously exercises a great influence in determining the seat and severity of the disease. Thus, in children born in a state of debility and emaciation, and in those who have long been deprived of the wholesome necessaries of life, we find all the tissues and organs of the body more or less soft and easily injured by external causes. This general diminution of cohesion is accompanied by universal pallor, a watery, scanty, and a plastic state of the blood. Such, also, is the case in advanced stages of tuberculous disease and scorbutus, the bones, as well as the other textures being found, in those who die of these diseases, soft, spongy, and infiltrated with a sero-albuminous, or sero-sanguinolent fluid." (Fasc. 5.)

We shall entirely pass over the observations upon softening produced by inflammation and by the obliteration of arteries, in order that we may have more room to notice the Softening by Gastric Acid; a subject peculiarly the author's own. There is no fact better established than that of the dissolution of the stomach and adjacent organs, by a secretion produced in the former. If any doubt could be entertained upon this point after the researches of John Hunter, Spallanzani, Adams, and Allan Burns, it must be dispelled from every candid mind by the experiments of Dr. Carswell. These we shall not enumerate, but proceed to the appearances presented by a stomach which has been acted upon by the gastric *acid*, as the author designates that secretion. That it *is* acid, is proved by its reddening litmus paper; and that the acid property is essential to its solvent action, is inferred from the fact that dissolution does not take place when the fluid has been neutralized. The situation of the softening (which may go on to erosion and perforation,) is always in the most depending part of the organ. This is generally the fundus, but unnatural states of neighbouring parts, (enlargement of the spleen and liver, for instance,) as well as the position of the body, must necessarily produce variations in this respect. The following description is so admirably faithful, that we should be sorry to disfigure by attempting to abridge it.

"The form of chemical softening of the coats of the stomach by the gastric acid presents several important varieties. If the softening be confined to the mucous membrane of the fundus, the form which it assumes is that of small or large patches. These are generally irregular, their bodies being formed by the mucous membrane, and the bottoms of each by the submucous coat; their edges, besides being irregular, are thin, soft, and somewhat transparent. If the softening has extended to the other coats of the stomach, the edges of these are beveled outwards, present a fringed appearance, or terminate in thin irregular prolongations which when water is poured upon them are seen to float like shreds of transparent coagulable lymph. Such are the forms of softening of the mucous membrane, so long as this membrane is smooth or stretched out by the contents of the stomach. But when this membrane is thrown into folds, or forms plicæ, the softening occurs no longer in patches, but presents those remarkable appearances described by M. Louis, as indicating the existence of pathological alterations. The forms of the softening in this case are those of stripes and bands of various dimensions occupying the situation of the plicæ. Wherever these stripes and bands exist, we find that the mucous membrane has been completely dissolved and the submucous coat laid bare. They have thus a bluish or silvery grey aspect, while the mucous membrane which they enclose may be of its natural colour, red, brown, or yellow, and appears in isolated patches of various forms and extent. It was the isolated and defined character of this form of softening which made it be considered as indisputably of a pathological nature. But the following explanation will show that it is a *post-mortem* lesion, and the consequence of the chemical action of the gastric acid. The mucous membrane possessing only in a very limited degree the power of diminishing its bulk, is always thrown into the form of plicæ when the muscular coat has contracted so as to diminish considerably the capacity of the stomach. When a quantity of gastric acid is collected on the surface of the mucous membrane in this state, it is obvious that the dissolvent property of this fluid will be exerted principally, if not exclusively, on the projecting borders of the plicæ, their lateral surfaces being protected from its action in consequence of their contiguity, or the mucous collected between them. Hence it follows, that when the stomach is removed from the body, emptied of its contents and spread out, the plicæ are effaced, and the stripes and bands, not before observed, make their appearance. That this is the manner in which this form of softening is produced is readily demonstrated by placing a quantity of gastric acid or digested food on the mucous membrane of a stomach in which the plicæ are well marked. In a few hours the appearances I have described will be conspicuous. The presence of gas in the stomach gives rise to another form of softening which requires to be noticed. The softening terminates in a well-defined abrupt margin, beyond which the mucous membrane is found to present (so far, at least, as the gastric acid is concerned) its natural colour and consistence. The regular and defined margin of the softening is determined by the gas acting as a foreign body, equalizing the distribution of the acid, and confining its operation to a circumscribed portion of the mucous membrane." (Fasc. 5.)

The colour of the part acted upon by the gastric acid is modified by the quantity of blood contained in the vessels. Under our own observation, it has been nearly always of a dull white, traversed by lines of a sooty appearance, evidently the remnants of blood-vessels, the contents of which had been discoloured by the acid. When the tissue is quite pale, as in infants and in leucophlegmatic subjects, the softened part, according to Dr. C., presents the same appearances as those described by Cruveilhier as gelatiniform softening, and as the result of a peculiar morbid process during life. This view is altogether denied by our author, who believes that the supposed instances of this disease are referrible to the mere chemical change. Upon this point we are of opinion that Dr. Carswell has not done full justice to Cruveilhier; though we believe the omission to have been unintentional. After reading the essay of our countryman, we might very easily imagine that the French pathologist had arranged under *Ramollissement gélatiniforme* changes which ought undoubtedly to be attributed to chemical action: whereas, he has described very fully a disorganization which he terms *Ramollissement pultacé,** and which corresponds exactly to that which Dr. Carswell has been so successful in explaining. Cruveilhier has moreover delineated this softening in a plate, the beauty and accuracy of which are not surpassed even by those in the work before us. Several distinctions are drawn by him between this alteration and the gelatiniform softening; and he adduces some special reasons for considering the latter to be a vital process, the most cogent of which are the alleged facts that it is occasionally found in the anterior parietes of the stomach, and that it occurs in individuals who have died after a very short illness, the symptoms of which were those of intense irritation in that organ. The argument derived from the symptoms may perhaps be neutralized by enquiring, with Dr. Carswell, how we are to account for these appearances being often absent in persons who die with similar signs of derangement, except by concluding that, in these instances, the gastric acid was wanting. We doubt, however, whether persons do die so suddenly of any other spontaneous structural disease of the stomach than that which Cruveilhier has described.

The occasional situation of the lesion in the *anterior* part of the organ, if a fact, is not to be disposed of very easily; but, with the exception of Cruveilhier's case, (of which he has given a delineation,) we know of no instance in which the lesion is specified as having occupied that situation, unless the much-controverted case of Miss Burns may be considered an example. In the mean time we see nothing unphilosophical in the idea, that there may be a spontaneous perversion of nutrition in the coats of the stomach, as well as in the brain or in any other organ, or in Cruveilhier's notion "que les maladies impriment à nos organes des altérations tout-à-fait identiques à celles que certains agens physiques et chimiques peuvent déterminer." Dr. J. Gairdner, who published, in the first volume of the Edinburgh Medico-Chirurgical Transactions, a very interesting paper entitled "Cases of Infantile Disease, in which Erosion and Perforation of the Alimentary Canal were found after Death," appears to hold a sort of middle opinion, viz. that in some cases the disease renders the tissue more susceptible of the solvent action. On consulting his tables which contain upwards of forty cases of perforation, we could not find one in which the lesion was in the fore part of the stomach. In conclu-

*Anat. Pathol. liv. x.

sion, we must remark that these changes are highly deserving of study, not only with reference to what we have just hinted at, but also in connexion with the action of deleterious substances. As yet we are not aware that the action of any irritating substance can imitate the process in question, be this a spontaneous softening or a chemical dissolution; but we must bear in mind that it does not necessarily preclude the idea of poisonous agency.

The fasciculus devoted to *Hemorrhage* contains an exceedingly well-executed compendium of modern researches upon the subject; and, though interspersed with many remarks which prove that the phenomena have passed under the author's own observation, yet is furnished with less of original matter than the preceding numbers. This is a necessary consequence of the advancement which had been effected in this department of pathology, previously to Dr. Carswell's researches. We imagine that there are few subjects upon which the minds of physicians are so well agreed as upon the nature of the different states of the system, or of parts of it, in which hemorrhage occurs; notwithstanding that, in any given case, considerable doubt may arise as to which of the acknowledged causes must be considered answerable for the lesion. Perhaps, also, it may be said, that there are few subjects upon which so much light has been thrown by modern investigation, from the time of Cullen downwards. At present, the greatest field for discovery is in the modifications which hemorrhages undergo according to the organs, and in their relation with nervous functions. The knowledge which we require upon these points has respect not so much to the immediate cause of the hemorrhage, as to the congestion or determination which precedes it.

Dr. Carswell classifies hemorrhages under two heads, according as they result from physical or from vital lesions. The first class comprehends all hemorrhages produced by solutions of continuity, and by mechanical obstacles of the circulation, whether in the heart or in the vessels. Under the second class are arranged those occasioned by, 1st, certain alterations in the functions of the capillaries, as in vicarious hemorrhage, and in the anormal vascular tissue of cephaloma, and in erectile tissue; 2dly, by diseased states of the blood, as in scorbutis, and in certain forms of purpura and typhus; 3dly, by debility, as in the depending parts of the body. We are somewhat surprised that, neither in this classification, nor in the remarks which follow it, is any mention made of that hemorrhage which results from plethora or hyperæmia, whether general or partial; a form of the disorder which meets us almost as frequently as any other, and upon the diagnosis of which such momentous questions in practice depend. Certainly it is not less established that hemorrhage is a common result of an excess of circulating fluid in the whole or in a part of the system, than that inflammation and dropsy are chargeable to such a condition. It is true that hemorrhage from partial plethora might seem fairly susceptible of arrangement under the alterations of the functions of the capillaries; yet it does not necessarily belong to those specified by the author, since it may be neither vicarious of a natural action, not dependent on a preternatural tissue.

In the course of some remarks upon the locality of sanguineous effusion, and particularly upon this occurrence within the cranium, the author departs from his usual course to speak of the connexion between paralysis of particular parts of the body and hemorrhage in particular divisions of the encephalon. His observation has led him to dispute the correctness of the statements that have been made respecting the correspondence of paralysis of the inferior extremities with effusion in the corpora striata, or in the parts level with or anterior to them; and an analogous correspondence of paralysis of the superior extremities with effusion in the thalami, or on a level with or posteriorly to them. Nor is he less unwilling to allow any necessary connexion between loss of speech and effusion into the anterior lobes of the brain. It has afforded us much satisfaction to find his experience upon this subject so confirmatory of our own. Considering the high rank occupied by the pathologists from whom the observations alluded to were derived, we should have been disposed to mistrust our own negative opinion, unless supported by such powerful authority as that of our distinguished countryman. We do not hesitate to copy his summary of the best established facts regarding the question at issue.

"1st. That the paralysis occupies the side of the body opposite to that of the brain or cerebellum in which the effused blood is situated.

"2d. That the paralysis affects only one side of the body when the effused blood is confined to one hemisphere of the brain, or one of the lateral lobes of the cerebellum.

"3d. That the paralysis exists on both sides of the body when the hemorrhage has taken place in both hemispheres of the brain, or both lateral lobes of the cerebellum; into the ventricles; the pons varolii the medulla oblongata; and on the surface of the brain.

"4th. That paralysis of both sides of the body may also take place when the hemorrhage is confined to one hemisphere of the brain or lateral lobe of the cerebellum, but is so extensive as to produce compression of the opposite hemisphere or lobe." (Fasc. 6.)

It may not be out of place to remind our readers that the researches of Andral have led him to very similar conclusions respecting the insufficiency of any pathological facts yet ascertained, to establish what parts of the brain are the respective organs of motific impulse for the superior and inferior extremities. Out of seventy-five cases of cerebral hemorrhage sufficiently circumscribed to serve for the illustration of the question, forty had been attended with hemiplegia, affecting both the arm and the leg. Of these forty, twenty presented the lesion either in the anterior lobe or in the corpus striatum; while, in nineteen, the lesion was either in the posterior lobe or in the thalami. In twenty-three out of the seventy-five, the paralysis was confined to the upper extremity; and, of these, eleven were caused by hemorrhage in the corpus striatum or in the anterior lobe, ten by the same lesion in the thalamus or in the posterior lobe, and two in the middle lobe. In twelve out of the seventy-five, the paralysis affected the lower extremity only; and, of these, ten were complicated with hemorrhage in the corpus striatum or in the anterior lobe, and two with hemorrhage in the posterior lobe or in the thalami. Undoubtedly, says Andral, there are distinct origins of motion for the arm and leg, but as yet we are unacquainted with their locality.*

An interesting remark is made upon venous hemorrhage from ulceration of a varix. In a case of this kind, hemorrhage from a very moderate-sized vein may be fatal in a few minutes, in consequence of the thickening of the parietes by chronic inflammation, and of their union with surrounding cellular tissue in a state of induration; circumstances which prevent the sides from being brought into contact, unless considerable pressure be exerted. Among some observations upon the effects of effusion upon the parts in which it occurs, a valuable case is related in proof of the capability which the brain possesses of bearing compression, when made gradually. A man fell upon the pavement, in a fit of intoxication, but suffered no material inconvenience, except a diminution of muscular strength and steadiness for three weeks afterwards; about which time he drank a strong stimulating potion, and soon afterwards became apoplectic and paralytic, and died on the following day. Dissection proved that six ounces of blood had been effused between the dura matter and arachnoid: it was partly fluid and partly coagulated, and red, or almost black; there was also false membrane on the internal surface of the dura matter, in which tissue several new vessels had been formed. These appearances proved that the effusion had been gradual, and that the compression had only produced serious mischief when the excitement of the circulation by the potion had hurried more blood into the cerebrum than could be tolerated, in addition to what had been effused. The author hints at analogous instances in the history of chronic abscesses, and in the slow accumulation of water in the ventricles, in illustration of the law, that the derangement produced by pressure is in a direct ratio with the rapidity with which the latter is effected.

The section upon the changes which the effused blood undergoes will be perused with much interest. It contains an excellent account of the two principal modes in which sanguineous effusions in the brain are disposed of: the first consisting of coagulation and conversion into a fibrous tissue, which is gradually reduced to a mere cicatrix; the second being the gradual transformation of the fibrin-

* Clinique Médicale, t. v. p. 358.

ous substance into a loose cellular tissue, containing serous fluid, which fluid increases, while the cellular membrane diminishes, till a complete cyst is formed; the removal of which may in time be affected by absorption of the fluid, and adhesion of the walls of the cyst, so as to form a cicatrix.

"Such appear to be the two modes which nature employs to accomplish the cure of a solution of continuity occasioned in the brain by an effusion of blood. That the paralysis generally diminishes during the progress of the curative process; that the degree of diminution which it undergoes corresponds sometimes with the more advanced stages of this process and that it completely disappears with the cicatrization of the original lesion, are facts well established. But the cases in which these relations are observed are far from being numerous, the same stage of the curative process—a cyst, being often found in cases of severe paralysis which had never undergone any sensible diminution; in others in which it had nearly disappeared, and in some few in which no traces of it were observable several years before death. Some of these differences with regard to the degree of the paralysis may depend on a difference in the seat of the original lesion, the extent of this, and the degree of injury done to the fibrous structure of the brain. But how far these or other circumstances will be found sufficient to remove the difficulties which beset this subject remains to be determined by future observation." (Fasc. 6.)

We must refer the reader to the description of the physical qualities of hemorrhage, according to the tissues and organs in which it occurs; and particularly to the researches upon pulmonary hemorrhage, and also those upon hemorrhage in the skin and cellular tissue. The latter would have been more complete, had the author alluded to that form of purpura, whether simple or hemorrhagic, which depends upon a plethoric and sthenic state of the system: whereas his remarks apply only to those forms which result from a deteriorated state of the blood. The plates in this fasiculous are admirably true to nature, and therefore highly instructive. That which illustrates the formation of hemorrhoidal tumors, and that which represents the extravasations sometimes produced in the stomach by irritating substances, are worthy of particular attention.

The fasiculus headed *Mortification* abounds in details of the highest value, and at the same time happily exhibits how wide an illumination may be shed over a great variety of facts, before obscure and unconnected, by the exposition of one or two simple principles. Thus, in following the author through his masterly description of the different changes which occur in that form of gangrene, or sphacelus, which results from inflammation in various tissues, the student will find them intelligible enough, when viewed as dependent on a cessation of the circulation; a state which, in some parts, is produced either by the pressure which the fluids, effused in consequence of the inflammation, exert upon the vessels or by the mere coagulation of the blood in them, and by their consequent obstruction. These changes may ensue either in the vessels of the mortified tissue itself, or of that from which it derives its nutriment. To take an instance in the morbid appearances presented by the cellular tissue in fatal cases of erysipelas phlegmonodes.

"They would seem to prove that the rapidly destructive effects of this form of inflammation depend in a great measure on the mechanical influence exercised by the effused fluids on the capillary circulation. These fluids must compress the neighbouring veins to a degree that will prevent the return of the blood poured into the capillaries, the function of nutrition must then cease to be accomplished, as is proved by the great diminution of cohesion which is observed to have taken place in the cellular tissue in the second stage of the disease; and as there is no tendency towards the formation of coagulable lymph, by the presence of which alone the inflammatory congestion can be arrested, the state of gangrene which this stage indicates must necessarily terminate in sphacelus. It is indeed well known that free mechanical division of the skin and cellular tissue, especially in the first stage of the disease, is the most effectual means that can be employed to arrest its progress;—a mode of treatment the obvious effect of which is to remove to a greater or less extent the distention of these tissues and the mechanical cause by which it is produced." (Fasc. 7.)

In furunculus and carbuncle, the nutrition of a portion of cellular membrane is cut off by the pressure of the surrounding tissue, tumefied and indurated both by the accumulation of blood and by the effusion of serum; a simple and satisfactory explanation of what is commonly called loss of vitality. In the pulmonary substance a similar set of changes may occur. In serous membranes, the circulation is arrested, not so much by lesion of their own vessels, as of those upon which the latter are entirely dependent for their supply of fluid: those, namely, which belong to the subserous cellular membrane, and which may be either obstructed or destroyed by inflammation and sphacelus. Mortification in fibrous membranes, cartilage, and bone, is always preceded by destruction of surrounding tissues. In the pleura, it is frequently caused by tubercles under that membrane. Many other instances are enumerated and explained in the most lucid manner in the first section, or that which treats of mortification as the result of inflammation. The remarks upon the state of the vessels in mortification, as observed in experiments upon the frog's foot, or in the mesentery of the rabbit, are concise, but present little novelty. Respecting the prevention of hemorrhage in gangrenous processes, the author expresses an opinion that, while it is occasioned in the smaller vessels by adhesive inflammation, it is dependent in the larger on the coagulation of the blood which they contain. Thus, it often happens that, in amputation of a limb, rendered necessary by the threatened spread of gangrene, the large vessels yield little or no blood, being obliterated by coagula.

The cessation of the circulation, in most of the cases to which we have hitherto alluded, has been occasioned by an arrest of the supply of food going to the tissue; but it may also result from an obstruction to the return. Thus, gangrene of the lower extremities may be the consequence of disease of the heart. This remote obstacle, however, will be greatly assisted in the production of gangrene by the effusion of serum which it has occasioned in the limb, and by the consequent pressure on the veins. Internal organs are likewise subject to gangrene from venous obstruction. A deposit of coagulable lymph or of tubercle may obstruct the venous circulation of a portion of lung, and cause it to sphacelate: and carcinomatous tumours may have the same effect in the liver. The following extract well describes the process in another internal organ.

"Mortification from a mechanical obstacle to the return of the venous blood, is well exemplified in intus-susception of the intestines. When the superior portion of intestine passes into the inferior, it carries along with it that part of the mesentery to which it is attached. If it does not suffer much compression, the invaginating process may go on to a great extent; but if it is compressed to such a degree that the return of the venous blood is obstructed, this stage of the disease is arrested, on account of the congestion of all the tunics of the invaginated portion. The congestion is not the consequence of inflammation; it is produced by compression, and in the following manner:—when the mesentery is put on the stretch by the descent of the superior into the inferior portion of the intestine, the veins belonging to it are compressed between the walls of both portions, just at the point where the invagination terminates superiorly. If adhesive inflammation takes place at this point, the peritoneal surfaces of both portions become united, and the veins obliterated. As the arteries are much less affected by pressure than the veins, they continue to pour in their blood into the invaginated portion; this fluid accumulates, and produces an extreme degree of congestion of the mucous and submucous coats, giving to them a deep red or almost black colour. In this state, however, the intestine is not deprived of its vitality. It is in a state of gangrene, but not of sphacelus; for its structure is still entire, and it may, when separated and evacuated, present, after having been macerated for some time so as to deprive it of the blood which it contains, the most perfect state of integrity of all its tunics. Occasionally, however, a portion of the whole of the invaginated intestine is found in a state of complete sphacelus, and is passed in the form of irregular spongy masses or shreds of a dirty ash-grey, brown, or black colour." (Fasc. 7.)

Of mortification by mechanical obstruction of the arteries, gangræna senilis is a familiar and striking illustration. The author very forcibly exposes the error of attributing this state to acute inflammation of the arteries; an opinion delivered in the "Leçons Orales" of M. Dupuytren.

Mortification may affect a part where neither inflammation has previously existed, nor the circulation been mechanically obstructed.

It may be the mere effect of *debility*: by which is meant such a change in the innervation and nutrition of the part as renders it incapable of removing an accumulation of blood, and apparently causes an alteration in the chemical qualities of this fluid. There is usually, however, some accidental congestion antecedent to the change in question, determined by such causes as pressure, friction, punctures, leech-bites, &c. To this species belongs the sphacelus of scorbutic patients, and that frightful disease of children called Stomacace gangrenosa, Necrosis infantilis, &c.; of which the reader will find a vivid description in this fasciculus. (Fasc. 7.) There is a section upon mortification from mechanical, chemical, and physical agents; and another upon mortification from certain poisons, such as the venom of certain animals, the products of disease or of putrefaction, and the ergot of rye; but they do not contain any thing sufficiently remarkable to induce us to dwell upon them.

This fasciculus contains a beautiful plate illustrative of gangrenous perforation of the intestines. The others strike us more by the artist-like manner in which they are executed, than by the information which they communicate.

The title of the last fasciculus yet published recalls some objections, which we have urged more than once. Under any view of the process whereby *Pus* is secreted, we should be unwilling to designate it as an "elementary form," but particularly if the principle be admitted, which the author takes great pains to establish, that it is always preceded by inflammation, and consequently that it is neither an elementary form nor an initiatory event.

Dr. Carswell, among his many other claims upon our approbation, presents that of being zealous in asserting the honours of British medicine. In the warmth of their admiration for the industry of the continental authors, writers in this country have of late years been too much inclined to ascribe all the important discoveries in pathology to a foreign origin; a notion which an exclusive perusal of continental publications would be very likely to engender. In the present fasciculus, by a careful and concise analysis of Mr. Hunter's researches, it is proved that this great pathologist was the first to effect a complete destruction of the old errors upon the formation of pus, and to establish a rational explanation of the process. It need scarcely be mentioned, that suppuration was in former times supposed to be a conversion of solid into liquid matter, by solution, corrosion, putrefaction, &c. The true nature of the process was first suspected by Dr. Simpson, of St. Andrew's, who, in 1722, compared a suppurating surface to "a kind of new gland." De Haen, in 1756, hinted that pus is a direct secretion from the blood; but this opinion, as is remarked by Professor John Thompson, was first embodied into a general doctrine in an inaugural essay by Dr. Morgan, in 1763.

"The principles of the new theory of the formation of pus which had been thus introduced, soon found several able supporters, among whom Mr. Hunter may be regarded as the first who furnished satisfactory evidence of their accuracy, by means of new facts and illustrations derived from his experiments on living animals, and his researches on suppurative inflammation and on pus." (Fasc. 8.)

After a statement of Mr. Hunter's conclusions, our author describes the changes in a suppurating part, according to the well-known microscopical researches of Kaltenbrunner and Gendrin. Besides the important observation of the actual conversion of the globules of blood into pus, these experimenters established that the change takes place, not merely when the blood is contained in the capillaries, but also when it has been effused into the surrounding tissue; whence it necessarily follows that the process is independent of capillary mechanism. The essential changes are, cessation of the circulation, coagulation, loss of colour and conversion of the fibrin into pus; but, in order that the blood, when coagulated, should undergo this transformation, it is essential, in Dr. Carswell's opinion, that the surrounding parts should be in a state of inflammation. In one experiment, after having injected a quantity of blood into the subcutaneous cellular tissue, Gendrin passed a seton through this tissue in order to excite inflammation; and the consequence was, that the effused blood was converted into pus. In another experiment, he injected an irritant liquid into a portion of vein included between two ligatures, and after re-admitting the blood, confined it in the same position; coagulation and suppuration were the result. These experiments certainly warrant the opinion that the changes of the blood were direct consequences of the inflammation; but they do not seem to us to prove that suppuration might not follow the coagulation induced by other causes. Now, there is no question that pus has often been found in tissues where there was not the slightest proof of inflammation having existed; as, for example, in coagula contained within the cavities of the heart: not to mention those anomalous purulent deposits which excited so much discussion a few years ago. In these cases, Dr. Carswell considers that the mere presence of pus as a foreign body has been often mistaken for a vital process of suppuration. His own experience has led him to believe that these deposits are never found unless inflammation and suppuration have existed in some parts of the system. The statement made by Andral and Maréchal, of their having discovered pus in the coagula of the heart, when no traces of suppuration could be detected elsewhere, he considers too vague to alter his belief; and he, moreover, thinks that the fluid found in such cases is not genuine pus, though it resembles that which is contained in concretions formed during inflammation of the internal membrane of the heart; a circumstance which tends to identify it as a product of inflammation.

It is certainly a fact deserving all the importance which Dr.

Carswell attaches to it, that, in nearly all the cases of suppurative inflammation with which the anomalous formations in question are complicated, the pus is situated in the veins of the part affected; in such cases it is easy to perceive how the morbid product may be carried into the blood. The cases alluded to are those connected with external suppurating sores, wounds, operations, fractures, phlebitis (either idiopathic or consequent to bloodletting), parturition, &c. &c. After admitting that pus is conveyed into the blood, we have to consider in what manner it produces the deposits in various tissues. Two opinions have been formed upon this subject: Velpeau, Maréchal, and Rochoux consider the deposits to be the consequences of the separation of the pus from the blood, and its subsequent accumulation in the capillaries or in the cellular tissue. According to others,—Dance, Blandin, and Cruveilheir,—they are the result of suppurative inflammation, induced by the pus acting as a foreign body, either in the capillaries or in the smaller veins. Both these opinions are pronounced by our author to be supported by numerous facts: but upon this subject we prefer that he should speak for himself.

"We have already seen that such deposits take place in coagula formed in the cavities of the heart, and it would be difficult to assign a reason why they should not take place in other organs, if circumstances occurred to interrupt the circulation through the capillaries. It is possible that the pus itself might effect this change in the capillary circulation, and occasion those circumscribed congestions so conspicuously seen in the lungs, and which constitute the first stage of the purulent deposits in these organs. The formation of the pus in these cases can be seen to take place in the same manner as in the coagula in the cavities of the heart. The blood with which the capillaries are distended, and which is sometimes effused at the same time into the surrounding cellular tissue, is coagulated, of a deep red colour, and forms globular masses varying from the size of a pea to that of a walnut, of considerable density. The first appearance of the pus in these masses is recognized by the presence of a number of pale yellowish-grey points, which increase in number and bulk until the whole of the blood is converted into a substance resembling in colour and consistence the fibrine of the blood. After this, the second stage is observed to follow the conversion of the fibrine into pus, and the formation of an abscess, the size of which is determined by the extent of the previous congestion. In some cases, however, the formation of the pus does not appear to be preceded, in the manner just described, by the separation of the fibrine into a solid mass It is occasionally found in drops in the coagulated blood, and therefore bearing a stronger analogy to what we have seen takes place in coagula in the cavities of the heart. A similar mode of formation of the purulent deposits has been observed in the subcutaneous and intermuscular cellular tissue, in the spleen, and in the liver. In this latter organ, however, these deposits present an appearance peculiarly characteristic of their origin, and which will be described in the plates in which they are represented. To these facts, in support of the opinion that purulent deposits are formed by the separation of pus from the blood, may be added those which disprove the existence of inflammation as their efficient cause, such as the absence of vascular congestion, of coagulable lymph, induration or softening of the surrounding tissues, and of any appreciable modification of function of the affected organ, more especially of such a nature as accompanies this pathological state, when it terminates in suppuration to the extent which the purulent deposits frequently occupy.

The opinion that the purulent deposits are the result of suppurative inflammation, induced in remote organs by the pus circulating in the blood, appears to me to rest also on the most conclusive evidence. In many cases, we meet with all the physical characters of inflammation; and, besides, the tissues in contact with the pus are softened, ulcerated, or covered with layers of coagulable lymph. There are sometimes pain, heat, and swelling in the joints in which the pus is found after death, and also in the situation of abscesses formed in the intermuscular and subcutaneous cellular tissue; and well-marked symptoms of pleurisy precede the formation of similar collections of pus in the cavity of the chest. Indeed we very often find coagulable lymph on the pleura, where it covers the purulent deposit in the lungs. I have stated that the inflammation which gives rise to the present form of purulent deposition is said by some pathologists to have its seat in the minute veins; that in fact these deposits not only have their remote origin in phlebitis, but that they are likewise the immediate consequence of the same morbid state. I have certainly not been able to satisfy myself of the accuracy of this opinion in cases of purulent deposits in the lungs or liver; and that it applies to those which form in the serous and synovial membranes cannot be admitted, unless phlebitis and inflammation are considered as synonymous terms." (Fasc. 8.)

The length of this foregoing extract precludes our quoting the concluding remarks upon this interesting quotation. They intimate that the theory which attributes the purulent deposits to the circulation of pus with the blood is confirmed by the symptoms which accompany their formation, and which indicate a sudden and general disturbance of all the functions of the economy: the resemblance which they bear to the effects of the injection of pus and putrid fluids into the veins of the inferior animals, is another corrobarative circumstance. The organs most subject to these accidents are the lungs and the liver; but recent researches do not authorize the opinion, once entertained, that they occur in the liver more frequently after injuries of the head than of the extremities. "Of all the organs of the body, the kidneys are least frequently the seat of purulent deposits."

We are by no means sure that Andral's statement respecting the presence of pus in the coagula of the heart, without the existence of inflammation in any part of the body, can be disposed of so readily as our author is inclined to think. But, allowing this statement to be incorrect, and that there is always a coexistent suppurative inflammation in some part, and that it is seated in the veins of that part, and moreover that pus enters the circulation, we do not see that the two methods above described are the only ones by which purulent deposits are effected, even if they be in any case adopted. With regard to those instances in which the deposits *are unaccompanied by inflammation of the tissue*, is it more probable that the pus was derived from a circumscribed phlebitis, through the agency of a mechanical mixture with the blood, and a subsequent separation, (in a quantity often disproportionate to that of its alleged source?)—or that the formation is attributable to a vital change in the constitution of the blood, occasioned by the pus introduced into it, (as in the analogous cases of injection into the veins of inferior animals,) which altered constitution becomes manifest by a secretion of a matter, similar to that with which it was infected, in tissues favourable to the process? When, in consequence of a morbid secretion injected into the veins of an animal, a secretion is formed in that animal with properties precisely similar to, and in far greater quantity than that which was introduced, shall we say that the morbid product is that which came from without; that it circulated with the blood, and was then separated from it, retaining all the while its identity? or shall we not rather infer that the matter injected occasioned a vital change in the composition of the blood, which in all probability corresponded with the state of the blood in the animal from which the infecting substance was obtained, and the result of which was an elaboration of a similar matter? To us there appears a striking analogy between the two cases. As far as the symptoms in each are concerned, the analogy is one of our author's own adducing; and we cannot help thinking that the symptoms which he himself enumerates, viz. "great prostration of strength, confusion, and stupor of the intellectual faculties, a dingy yellow colour of the skin, fœtor of the breath, meteorism, and petechiæ," are more likely to be the effects of such a change as we have hinted at, than of a mere admixture of pus with the blood. As an additional support to our suggestion, we may allude to the fact that purulent formations often take place after the removal of the part in which suppuration had been going on for some time. If the deposits were identical with the matter derived from the seat of the suppuration, we could scarcely expect them to occur precisely when the source is cut off. We think that they may be more rationally accounted for, as resulting from a sort of vicarious secretion, analogous to the anomalous deposits of bile or urinary salts, when the liver or the kidneys have become incapable of their function. Perhaps, Dr. Carswell would urge in reply to this suggestion, that he has proved inflammation to be a necessary antecedent to purulent fomations, and consequently that, if pus is formed where no inflammation exists, it must have been derived from some other part where inflammation *did* exist. But we are not yet prepared to admit that inflammation *is* essential; and although the experiments of Gendrin, and a vast number of pathological facts suffiently prove that inflammation was in those cases an exciting cause of the suppurative process, it does not follow that, in other cases, the process might not own some other excitant. Indeed, the very deposits of which we have been treating afford, in our opinion, and for the reasons which we have stated, strong presumptive evidence that other causes than inflammation have been in action.

As to the opinion that, in those cases in which the anormal deposits *are* attended with unequivocal marks of inflammation, the cause of the inflammation was pus in the capillaries, acting as a foreign body; while we do not undertake to deny the possibility of such an occurrence, it seems worthy of consideration whether the inflammation may not be the consequence of a change in the blood occasioned by the purulent matter conveyed into it.

But it is time to conclude. It has been both our duty and a gratification to us, in our critical capacity, to express the strongest approbation of Dr. Carswell's researches. To all who feel an interest in the study of their profession, these faithful Illustrations present objects of instructive contemplation; whilst to those who do not possess the advantage of residing near a large hospital, or whose opportunities of examining bodies after death are rare, such a conspectus of morbid anatomy, so vividly illustrated, is literally invaluable. The gratitude not only of speculative pathologists but of practitioners of every degree, is indeed due to Dr. Carswell for labours, of which the direct tendency is to improve the knowledge of the nature and treatment of a large class of maladies; and we may justly add, that his splendid work, the result of many years of laborious observation, reflects honour upon British pathology.

---

